# Normal pregnancy in a patient with β-thalassaemia major receiving iron chelation therapy with deferasirox (Exjade®)

**DOI:** 10.1111/j.1600-0609.2010.01569.x

**Published:** 2011-03

**Authors:** Dimitra Vini, Philippos Servos, Marouso Drosou

**Affiliations:** Thalassaemia Unit, Hospital of NikaiaAthens, Greece

To the Editor:

Improvements in managing β-thalassaemia major have allowed many patients to survive beyond puberty. Fertility can, however, be impaired as a result of iron overload-related hypogonadism (1), although assisted reproductive techniques and advances in treating iron overload have increased the number of successful pregnancies in such patients (2–9). During pregnancy, patients may require additional blood transfusions to treat complications such as pre-eclampsia or severe anaemia, increasing the need for effective iron chelation therapy. Clinical data are limited regarding the use of chelation therapy during pregnancy, and it is unclear whether these agents pose any risk to the developing foetus. However, there are case reports of successful pregnancies following treatment with deferoxamine (Desferal®; Novartis Pharmaceuticals Corp, East Hanover, NJ, USA) (10, 11). This case study discusses a patient who became pregnant while receiving deferasirox (Exjade®; Novartis Pharmaceuticals Corp), the once-daily, oral iron chelator. As deferasirox use is contraindicated during pregnancy, no recommendation can be made on the safety of iron chelation in pregnancy.

The 35-yr-old female patient (born June 1972) was diagnosed with β-thalassaemia major at 8 months of age. She has received red blood cell (RBC) transfusions since diagnosis and was treated with continuous deferoxamine from the age of 6 yr. Due to poor compliance and subsequent lack of efficacy, she was switched to deferiprone monotherapy (Ferriprox®; Apotex Inc., Toronto, ON, Canada) aged 29 yr. She received deferiprone for 2 yr, before switching to deferoxamine and/or deferiprone because of a lack of efficacy; she remained on this regimen until August 2006. The patient had her first menstruation at the age of 12 and had regular menstrual cycles until she was 30 yr old, when hormonal replacement therapy was required. She was splenectomised aged 27 yr. Obstetric history included two planned abortions and one successful pregnancy by *in vitro* fertilisation. She did not receive any chelation therapy during this pregnancy.

The patient was switched to deferasirox in August 2006 following enrolment into a clinical trial; at this point, she was receiving two RBC units every 20 d. Prior to enrolment, her compliance with deferoxamine had been poor, which led to substantially elevated body iron levels; the patient's clinical characteristics at this time are shown in Table 1. Deferasirox treatment was initiated at a dose of 18.9 mg/kg/d. Before starting deferasirox, a serum β-hCG pregnancy test was performed and proved negative. The dose was increased to 23.6 mg/kg/d after 3 months (November 2006) because of slow decrease in serum ferritin and increased weight. A mild skin rash was initially observed, although this resolved spontaneously without discontinuing deferasirox treatment. The patient also experienced persistent constipation for which she received lactulose (Duphalac®; Solvay S.A., Brussels, Belgium).

**Table 1:** Patient's clinical characteristics when switching to and stopping deferasirox treatment

Characteristic	Baseline values when switching to deferasirox	Values when deferasirox stopped (confirmation of pregnancy)
Haemoglobin (g/dL)	9.8	10.1
Haematocrit (%)	29.5	30.7
Serum ferritin (ng/mL)	3681	2431
Weight (kg)	53	55
AST (U/L)	19	18
ALT (U/L)	38	33
Serum creatinine (mg/dL)	0.7	0.4

AST, aspartate aminotransferase; ALT, alanine aminotransferase.

During a random examination by her gynaecologist on 24 December 2006, the patient was confirmed to be 22 wk pregnant; her last menstruation occurred 4 August 2006. During this period, the patient was receiving hormone replacement therapy. This second pregnancy was spontaneous. Deferasirox treatment was stopped immediately, at which point serum ferritin levels had decreased by >1000 ng/mL (Table 1). From the 17th week until the confirmation of pregnancy, the patient's haemoglobin level decreased (average 7.5–8.0 g/dL). Once deferasirox was withdrawn, the patient's constipation resolved completely, suggesting it may have been treatment related. Cardiac function, as assessed by Triplex and Doppler (General Electric Logiq), was normal during follow-up. According to Doppler examination, both dimensions and output were normal. Close monitoring of development via ultrasound was performed monthly throughout the pregnancy.

During pregnancy, the patient received an average of 5 RBC units/month until the end of April 2007. Serum ferritin levels after stopping chelation (during 2007) were as follows: January, 2721; February, 2431; March, 2357; April, 2311; May, 2743 and June, 2264 ng/mL. Since serum ferritin levels did not increase, the foetus may have acted as a natural chelator. Diabetes of gestation, which was also noted during her first pregnancy, was observed at 31 wk and required insulin treatment; the investigator considered this to be serious but not related to deferasirox treatment. A caesarean section was performed in May 2007 at 37 wk gestation. The baby was a normal female: weight 2.59 kg (5.7 lbs), height 47.3 cm, head circumference 33 cm and Apgar score normal (8 after 1 min). Birthweight, height and Apgar score were comparable to that of the child's older sibling, who is also female: birthweight 2.40 kg (5.3 lbs), height 51 cm, head circumference 32 cm and Apgar score normal.

In conclusion, this is the first report describing the use of deferasirox during pregnancy. In this case, treatment up to 22 wk gestation was well tolerated and did not prevent delivery of a healthy baby. The child is now 4 yr old and is developing normally. It should be noted that the use of deferasirox is contraindicated in pregnant women based on the approved product label.
